# Is There a Role for Sonde Enteroscopy in Patients with Obscure Gastrointestinal Bleeding? A Comparison with Capsule Endoscopy

**DOI:** 10.4021/gr2009.04.1284

**Published:** 2009-03-20

**Authors:** Samuel N Giordano, Jeff Dilisi, Kuldip Banwait, Daniel Wild, Anthony Infantolino, Lenore Miranda, Mitchell Conn

**Affiliations:** a Division of Gastroenterology and Hepatology, Thomas Jefferson University Hospital, 132 S. 10th Street, 480 Main Building, Philadelphia, PA 19107, USA

**Keywords:** Sonde, capsule endoscopy, Obscure gastrointestinal bleeding, Enteroscopy

## Abstract

**Background:**

In the 1980’s and 1990’s combined Push and Sonde Enteroscopy was the primary endoscopic tool used to evaluate the small intestine in patients with obscure gastrointestinal bleeding (OGIB). It was available in only a few centers due to the technical difficulties associated with its use. The introduction of wireless capsule endoscopy in 2001 revolutionalized small bowel endoscopic imaging making Sonde enteroscopy a rarely used procedure despite the lack of studies comparing the efficacy of the two modalities. The aim of this study was to restrospectively compare the findings of Sonde enteroscopy with capsule endoscopy in patients with OGIB.

**Methods:**

Design: One hundred patients who underwent Sonde enteroscopy and 101 patients who underwent capsule endoscopy were retrospectively studied. Setting: All patients had their procedures completed by physicians within the same gastroenterology practice. Patients: All patients who underwent either Sonde enteroscopy or capsule endoscopy were enrolled. Interventions: None. Main outcome measurements: Outcome was defined as the number of patients in which a distinct bleeding site could be identified.

**Results:**

A total of 100 patients underwent Push and Sonde enteroscopy and a potential bleeding site was identified in 55 (55%) patients. A total of 101 patients underwent capsule endoscopy and a potential bleeding site was identified in 60 (59%) patients. A one-tailed P value showed no statistically significant difference in the diagnostic yield between the procedures.

**Conclusions:**

Capsule endoscopy is at least as efficacious as Push/Sonde enteroscopy in evaluating patients with OGIB. We can comfortably retire Sonde enteroscopy as a diagnostic tool.

## Introduction

Obscure gastrointestinal bleeding (OGIB) is defined as bleeding in which the source remains unknown following initial evaluation with upper endoscopy and colonoscopy [1]. OGIB accounts for 3% to 5% of cases of gastrointestinal bleeding [2, 3]. The majority of these patients are ultimately found to be bleeding from the small intestine.

Complete endoscopic examination of the small intestine has been difficult due to its length and tortuosity. Push enteroscopy utilizing pediatric and adult colonoscopes and specialized small bowel enteroscopes have been used to examine the proximal small intestine. Diagnostic yield with push enteroscopy has ranged from 26% to 75% in several studies [4-9]. In the late 1980’s, Sonde enteroscopy was introduced and used to visualize the small bowel beyond the reach of the push enteroscope. Combined Push/Sonde enteroscopy resulted in a diagnostic yield of 40%-50% [10-12]. Despite these favorable results, Sonde enteroscopy was not universally accepted and only performed at a few institutions. The procedure was perceived as draconian associated with patient discomfort, labor intensive requiring hours of physician time, purely diagnostic lacking therapeutic capabilities and variable in its ability to visualize the most distal small intestine.

In 2001 when small bowel capsule endoscopy was introduced, it rapidly supplanted Sonde enteroscopy and became the endoscopic procedure of choice for visualization of the small bowel. Studies confirmed its superiority over push enteroscopy. The diagnostic yield of capsule endoscopy has been reported to range from 55% to 68%, compared to push enteroscopy, which in randomized studies, had a diagnostic yield of 28%-32% [13-15]. In 2003, Dr. Jerry Waye proclaimed that Sonde Enteroscopy can be laid to rest [16] despite the lack of any comparison between the two techniques appearing in the literature.

In 2004, Double Balloon Enteroscopy was introduced offering a therapeutic modality to small bowel pathology. Early reports have demonstrated its diagnostic and therapeutic benefits [17, 18]. It would seem appropriate then, with the development of both capsule endoscopy and double balloon enteroscopy that Sonde Enteroscopy can be laid to rest along side the Gavin Balloon and the gallstone lithotripter. However, without a comparison study we may overlook any benefits or advantages Sonde enteroscopy may have had. One could argue that Sonde enteroscopy allowed direct physician involvement and afforded some level of control of the endoscope during visualization and withdrawal when compared with the capsule. Sonde enteroscopy had air insufflation and water irrigation capabilities. In light of this, the aim of our study is to compare our experience with Sonde enteroscopy and capsule endoscopy in patients with obscure intestinal bleeding at our institution.

## Materials and Methods

Prior to the development of capsule endoscopy, the gold standard at our institution was to perform Sonde enteroscopy to evaluate patients with OGIB. Our technique of performing Sonde enteroscopy has been previously reported [19]. All patients we evaluated undergoing Sonde enteroscopy also had a push enteroscopy performed on the same day. Extent of insertion of the Sonde Enteroscope was estimated by imaging with a plain abdominal X-ray prior to removal of the instrument. Potential bleeding sites were defined as abnormalities known to cause bleeding such as vascular ectasias, ulcers, erosions and tumors. Lymphangiectasias, lipomas, and diverticulum were not considered as potential causes of bleeding. One endoscopist performed all Push/Sonde enteroscopies (PSE) during the time period of June 1993 through February 2001. A total of 100 consecutive patients who underwent PSE were included in this study. Since December 2001, we have been performing capsule endoscopy (Given SB PillCam) for the evaluation of patients with obscure gastrointestinal bleeding. The technique for performing and reading capsule endoscopy studies has been previously reported [20, 21]. The majority of patients referred to our hospital for capsule endoscopy have already undergone extensive endoscopic evaluation prior to our evaluation. The first 101 patients undergoing capsule endoscopy for obscure GI bleeding from December, 2001 to January, 2003 were included in our study. Relevant data and history on these patients was collected prior to the procedure, including the age and gender of the patient, clinical presentation, previous endoscopic evaluation(s), blood transfusion requirements, and procedure findings.

We performed a retrospective analysis between patients who underwent Push/Sonde enteroscopy compared with those undergoing capsule endoscopy. A two-tail *t*-test analysis was performed comparing PSE and Sonde alone to capsule endoscopy regarding these variables and each procedure’s diagnostic yield. The study was reviewed by the IRB committee at our institution and was approved.

## Results

A total of 100 patients who underwent PSE during the time period of June 1993 through February, 2001 were included in the retrospective chart review. The mean age of these patients was 67.3 years (range 30-87). Fifty-five percent of the patients were women and 45% were men. Among these patients, 46% presented with melena, 39% presented with occult bleeding, and 15% with hematochezia (Table 1). Seventy-three percent of these patients required prior blood transfusions and this group averaged 7.1 procedures before Sonde while the 27% not requiring transfusions averaged 4.9 procedures prior to Sonde.

**Table 1:** Overview of demographics and presenting complaints of PSE and M2A groups

	PSE (N = 100)	M2A Capsule (N = 101)
Men no. (%)	45 (45)	45 (44)
Women no. (%)	55 (55)	55 (56)
Age-yr	67.3 (30-87)	65.2 (13-87)
Chief presenting complaint		
Melena-no.(%)	46 (46)	27 (27)
Occult Bleeding-no. (%)	39 (39)	67 (67)
Hematochezia-no. (%)	15 (15)	6 (6)
Patients requiring previous transfusions-no. (%)	73 (73)	62 (62)

M2A, mouth-to-anus.

It was estimated that Sonde enteroscopy visualized into the ileum in 50% of the patients, with the distal ileum being visualized in 7%. Combined PSE identified 60 potential bleeding sites in 55 patients with an overall yield of 55%. Findings included: vascular ectasias in 38 patients (69.09%); active bleeding in 2 patients (3.64%); ulcers/erosions in 13 patients (23.63%); and tumors in 7 patients (12.72%); (Table 2).

**Table 2:** Comparison of findings between PSE and M2A

	Sonde Enteroscopy (n = 100)	Push/Sonde Enteroscopy (n = 100)	M2A Capsule (n = 101)
Patients identified with potential bleeding site	43% (n = 43)	55% (n = 55)	59% (n = 60)
Vascular Ectasias	60.46% (n = 26)	69.09% (n = 38)	56.67% (n = 34)
Active Bleeding	4.65% (n = 2)	3.64% (n=2)	15% (n=9)
Ulcer/Erosion	20.90% (n = 9)	23.63% (n = 13)	11.67% (n = 7)
Tumor	9.34% (n = 4)	12.72% (n = 7)	5% (n = 3)
Hemangioma	0% (n = 0)	0%( n= 0)	1.67% (n = 1)
Crohn's Disease	0% (n = 0)	0%(n = 0)	10% (n = 6)

M2A, mouth-to-anus.

Sonde enteroscopy alone identified a potential bleeding site in 43 patients, resulting in an overall yield of 43%. Among the 43 patients who had diagnoses identified by Sonde enteroscopy alone, 26 patients had vascular ectasias (60.46%); 2 had active bleeding (4.65%); 9 had ulcers (20.90%); 4 had tumors (9.34%); and 2 had erosions (4.65%). The location of these findings can be found in Table 3.

**Table 3:** Location of Sonde Enteroscopy Findings

Finding	Total findings	Duodenum	Jejunum	Ileum
Vascular ectasias	26	7.69% (n = 2)	73.07% (n = 19)	19.23% (n = 5)
Ulcers	9	0% (n = 0)	88.89% (n = 8)	12.11% (n = 1)
Tumors	4	0% (n = 0)	75% (n = 3)	25% (n = 1)
Erosions	2	0%(n = 0)	100% (n = 2)	0% (n=0)
Active Bleeding	2	0% (n = 0)	100% (n = 2)	0% (n = 0)

A total of 101 patients undergoing capsule endoscopy for the evaluation of OGIB from December, 2001 through January, 2003 were examined. The mean age of presentation for patients undergoing this procedure was 65.2 years (range 13-89), of which, 56% were women and 44% were men. Patients presented with melena, occult bleeding, and hematochezia were 27%, 67%, and 6% respectively. Sixty-two percent of these patients required blood transfusions prior to capsule endoscopy. The majority of patients for capsule endoscopy were referred from outside institutions as a result the number of procedures prior to capsule endoscopy was not available.

The capsule successfully visualized the entire small intestine and passed into the colon in 88.7% of the patients. All capsule passed spontaneously, there were no patients with retained capsules. A potential bleeding site was identified in 60 patients (59%). These findings included vascular ectasias in 34 patients (56.67%); active bleeding in 9 patients (15%); suspected Crohn's disease in 6 patients (10%); ulcers in 7 patients (11.67%); tumors in 3 patients (5%); and an hemagioma in 1 patient (1.67%).

Overall, there was no statistical difference in diagnostic yield between PSE and capsule endoscopy (P > 0.05). Capsule endoscopy visualized the small intestine more completely than push/sonde reaching the colon in 88.7% of patients. As expected, PSE never reached the colon. When the specific causes of bleeding were compared in the PSE group versus the capsule group, the capsule identified active bleeding more frequently than PSE, P < 0.05. No diagnoses of Crohn’s disease or hemangioma were made in the PSE group. More patients in the PSE group presented with active bleeding, i.e. hematochezia 15% versus 6% in the capsule group.

## Discussion

The aim of our study was to compare capsule endoscopy to PSE for the evaluation of OGIB. This comparison is important as capsule endoscopy has replaced combined PSE as the gold standard for evaluating patients with OGIB; however, a direct comparison has not been published in the literature. We found that the diagnostic yield of the PSE and capsule endoscopy was not significantly different in our study despite the fact that the capsule was found to examine a greater extent of the small intestine. The lack of difference in these findings can be explained in several ways. At the time of this study, capsule endoscopy was still a relatively new technology and therefore an initial learning period may have been required prior to optimizing its yield. In addition, more than one gastroenterologist contributed readings of capsule studies compared to PSE studies, which were all performed by only one gastroenterologist at our institution. Another explanation is that the distribution of bleeding sites in the small intestine may have a proximal predilection, with most of the OGIB sources being in the duodenum and jejunum, which are often within the reach of push/Sonde enteroscopy [2, 22]. Direct physician involvement in performing both push and Sonde enteroscopy may enhance its diagnostic yield, when compared with the observational nature of the capsule. These explanations could certainly explain why there was no overall difference in the diagnostic yield of PSE when compared to capsule for the diagnosis of OGIB.

We acknowledge certain weakness in our study. First this is a retrospective review of two different populations of patients. These two groups were different in the manner in which they presented for further endoscopic evaluation of obscure gastrointestinal bleeding. The patients in the PSE group presented more often with overt bleeding, documented as melena and hematochezia than the patients in the capsule group, who presented most often with occult bleeding. There has been a clear change in referral pattern since the introduction of capsule endoscopy. Sonde enteroscopy was reserved for the clinically more significant bleeding whereas clinically less significant bleeders are now being referred for capsule studies. If the PSE group is a higher risk group, then the diagnostic yield could approach that seen in the capsule group, not because it is as sensitive, but because the patients are more likely to have abnormalities.

A greater number of patients with active bleeding were identified by capsule endoscopy compared to PSE (P < 0.05), despite the fact that more patients presented with active bleeding in the PSE group. One explanation for this discrepancy might be that capsule studies are sedation free. Consequently, the capsule captures the appearance of the bowel mucosa in its natural state, rather than under the drug effects of conscious sedation, which can lead to attenuation of bleeding secondary to the vasoconstrictive effects.

Our findings suggest that capsule endoscopy is at least as efficacious as PSE in evaluating patients with OGIB. In addition, capsule endoscopy was shown to visualize more of the small intestine. Capsule endoscopy may also be better at diagnosing Crohn’s Disease than PSE, as no cases were observed in the PSE group compared to 10% in the capsule group. This probably reflects the capsule’s ability to more consistently visualize the distal ileum where Crohn’s Disease is most likely to occur. Recent studies support this evidence by demonstrating the diagnostic yield of capsule endoscopy in the diagnosis of Crohn’s disease compared to other modalities [23-25]. Sonde enteroscopy is no longer used at our institution given the capsule’s safety, avoidance of sedation, ease of use and overall effectiveness. With the introduction of Double Balloon Enteroscopy and its’ proven diagnostic and therapeutic abilities, we now utilize capsule endoscopy as an initial diagnostic tool for OGIB followed by Push or Double Balloon enteroscopy if findings on capsule study warrant its use. We can now comfortably lay to rest Sonde enteroscopy. May it rest in peace.
